# Long-term outcomes of neoadjuvant trastuzumab emtansine + pertuzumab (T-DM1 + P) and docetaxel + carboplatin + trastuzumab + pertuzumab (TCbHP) for HER2-positive primary breast cancer: results of the randomized phase 2 JBCRG20 study (Neo-peaks)

**DOI:** 10.1007/s10549-024-07333-7

**Published:** 2024-05-20

**Authors:** Toshimi Takano, Norikazu Masuda, Mitsuya Ito, Kenichi Inoue, Yuko Tanabe, Kousuke Kawaguchi, Hiroyuki Yasojima, Hiroko Bando, Rikiya Nakamura, Takashi Yamanaka, Kazushige Ishida, Tomoyuki Aruga, Yasuhiro Yanagita, Eriko Tokunaga, Kenjiro Aogi, Shinji Ohno, Hiroi Kasai, Tatsuki R. Kataoka, Satoshi Morita, Masakazu Toi

**Affiliations:** 1 Department of Breast Medical Oncology, The Cancer Institute Hospital of JFCR, Tokyo, Japan; 2 Department of Breast and Endocrine Surgery, Nagoya University Graduate School of Medicine, 65 Tsurumai-Cho, Showa-Ku, Nagoya, Aichi 466-8550 Japan; 3 Department of Breast Surgery, Hiroshima City Hiroshima Citizens Hospital, Hiroshima, Japan; 4 Division of Breast Oncology, Saitama Cancer Center, Saitama, Japan; 5 Department of Medical Oncology, Toranomon Hospital, Tokyo, Japan; 6 Department of Breast Surgery, Kyoto University Hospital, Kyoto, Japan; 7 Department of Surgery, Breast Oncology, NHO Osaka National Hospital, Osaka, Japan; 8 Breast and Endocrine Surgery, Faculty of Medicine, University of Tsukuba, Tsukuba, Japan; 9 Division of Breast Surgery, Chiba Cancer Center, Chiba, Japan; 10 Department of Breast Surgery and Oncology, Kanagawa Cancer Center, Yokohama, Japan; 11 Department of Surgery, Iwate Medical University, Morioka, Japan; 12 Surgery (Breast), Tokyo Metropolitan Komagome Hospital, Tokyo, Japan; 13 Department of Breast Oncology, Gunma Prefectural Cancer Center, Ota, Japan; 14 Department of Breast Oncology, NHO Kyushu Cancer Center, Fukuoka, Japan; 15 Department of Breast Oncology, NHO Shikoku Cancer Center, Matsuyama, Japan; 16 Clinical Research, Innovation and Education Center, Tohoku University Hospital, Sendai, Japan; 17 Department of Diagnostic Pathology, Iwate Medical University, Morioka, Japan; 18 Department of Biomedical Statistics and Bioinformatics, Kyoto University Graduate School of Medicine, Kyoto, Japan; 19 Tokyo Metropolitan Komagome Hospital, Tokyo, Japan; 20 Present Address: Department of Breast Surgery, Graduate School of Medicine, Kyoto University, Kyoto, Japan

**Keywords:** De-escalation, Long-term outcomes, Neoadjuvant therapy, Pertuzumab, Response-guided treatment, Trastuzumab emtansine

## Abstract

**Purpose:**

The randomized phase 2 Neo-peaks study examined usefulness of neoadjuvant trastuzumab emtansine + pertuzumab (T-DM1 + P) following docetaxel + carboplatin + trastuzumab + pertuzumab (TCbHP) as compared with the standard TCbHP regimen. We previously reported that pCR rate after neoadjuvant therapy tended to be higher with TCbHP followed by T-DM1 + P. We conducted an exploratory analysis of prognosis 5 years after surgery.

**Methods:**

Neoadjuvant treatment with TCbHP (6 cycles; group A), TCbHP (4 cycles) followed by T-DM1 + P (4 cycles; group B), and T-DM1 + P (4 cycles; group C, + 2 cycles in responders) were compared. Group C non-responders after 4 cycles were switched to an anthracycline-based regimen. We evaluated 5-year disease-free survival (DFS), distant DFS (DDFS), and overall survival (OS).

**Results:**

Data from 203 patients (50, 52, and 101 in groups A–C, respectively) were analyzed. No significant intergroup differences were found for DFS, DDFS, or OS. The 5-year DFS rates (95% CI) were 91.8% (79.6–96.8%), 92.3% (80.8–97.0%), and 88.0% (79.9–93.0%) in groups A–C, respectively. TCbHP followed by T-DM1 + P and T-DM1 + P with response-guided addition of anthracycline therapy resulted in similar long-term prognosis to that of TCbHP.

**Conclusions:**

In patients who achieved pCR after neoadjuvant therapy with T-DM1 + P, omission of adjuvant anthracycline may be considered, whereas treatment should be adjusted for non-pCR patients with residual disease. T-DM1 + P with response-guided treatment adjustment may be useful for minimizing toxicity.

**Trial registration number and date of registration:**

UMIN-CTR, UMIN000014649, prospectively registered July 25, 2014. Some of the study results were presented as a Mini Oral session at the ESMO Breast Cancer 2023 (Berlin, Germany, 11–13 May 2023).

**Supplementary Information:**

The online version contains supplementary material available at 10.1007/s10549-024-07333-7.

## Introduction

Human epidermal growth factor receptor 2–positive (HER2 +) breast cancer is an aggressive phenotype that has a poor prognosis [1]. Current treatment guidelines for this type of breast cancer recommend use of multidrug chemotherapy (i.e., a sequential combination of an anthracycline-containing regimen and a taxane, or simultaneous use of a taxane and platinum) combined with anti-HER2 agents [2, 3].

Dual HER2 blockade by the combined use of anti-HER2 agents with different mechanisms is likely to improve the efficacy of treatments for HER2 + breast cancer [4]. In the neoadjuvant setting, dual HER2 blockade with trastuzumab and pertuzumab combined with docetaxel has been shown to significantly increase pathological complete response (pCR) rate, with no significant differences in tolerability compared with trastuzumab plus docetaxel [5].

Trastuzumab emtansine (T-DM1), an antibody–drug conjugate (ADC) of trastuzumab with the microtubule inhibitor emtansine, has been developed and is being investigated as part of neoadjuvant therapy for HER2 + breast cancer. Its efficacy and safety when used in combination with pertuzumab have been evaluated to determine whether this type of dual HER2-targeted therapy could replace traditional systemic chemotherapy, thereby eliminating the toxicity associated with chemotherapeutic drugs. A systematic review has shown that addition of pertuzumab to either trastuzumab or T-DM1 with or without chemotherapy increases pCR rate in a neoadjuvant setting, with increased risk of tolerable toxicity and no significant differences in cardiac toxicity [6]. However, the results of the phase 3 study showed that significantly more patients achieved pCR with traditional chemotherapy plus dual HER2 blockade (with trastuzumab and pertuzumab) than with the T-DM1 + pertuzumab (T-DM1 + P) regimen [7].

To further investigate potential benefits of the T-DM1 + P regimen, with the aim of minimizing treatment-related toxicity and increasing pCR rate, we conducted the Neo-peaks study to compare the following 3 neoadjuvant therapy regimens: docetaxel + carboplatin + trastuzumab + pertuzumab (TCbHP), TCbHP followed by trastuzumab emtansine + pertuzumab (T-DM1 + P), and T-DM1 + P (response guided) [8]. Our results showed that pCR rate was numerically higher in patients who received the TCbHP followed by T-DM1 + P regimen (71.2%) than in those who received the standard TCbHP (56.9%) or T-DM1 + P regimen (57.4%). Additionally, in the patients with estrogen receptor–positive (ER +) disease, pCR rate was significantly higher in those who received TCbHP followed by T-DM1 + P compared with those in the other groups (69.0% versus 43.3% for standard TCbHP and 50.8% for T-DM1 + P).

Although pCR after neoadjuvant therapy is an important predictor of long-term prognosis and is used as the primary endpoint of many neoadjuvant studies, the relation between pCR achievement and prognosis after neoadjuvant chemotherapy may vary depending on cancer subtype; for example, pCR appears to be a suitable surrogate endpoint for patients with hormone receptor (HR)-negative and HER2 + disease but not for those with HR-positive and HER2 + disease [9].

We analyzed long-term survival data from Neo-peaks to determine whether pCR after neoadjuvant chemotherapy is associated with long-term survival in patients who received each of the 3 different regimens used in that study, as well as in subgroups of patients with different ER status. We also explored outcomes in patients who received the chemotherapy-free regimen and the potential usefulness of treatment adjustment by means of administration of additional anthracycline therapy based on individual patients’ response to T-DM1 + P.

## Methods

### Study design and dataset

Neo-peaks (Japan Breast Cancer Study Group study 20; UMIN-CTR: UMIN000014649) was a randomized, phase 2, open-label, 3-arm study for which patients receiving treatment at 17 centers across Japan were enrolled between August 2014 and February 2016 [8]. For the present follow-up study, data collected up to November 2021 were analyzed to evaluate long-term outcomes and safety. All patients who participated in the Neo-peaks study were included in the present study, excluding any patient who withdrew consent for her data to be used in follow-up studies.

### Neoadjuvant therapy

Details of the Neo-peaks study, including the inclusion and exclusion criteria, have been published previously [8]. Briefly, patients with HER2 + primary, operable breast cancer (cT1c–cT3, cN0–cN1, cM0; target lesion ≤ 7 cm) were randomized in a 1:1:2 ratio to receive one of the following options for neoadjuvant therapy: 6 cycles of TCbHP (group A), 4 cycles of TCbHP followed by 4 cycles of T-DM1 + P (group B), or 4 cycles of T-DM1 + P (group C); subsequently, based on the degree of tumor shrinkage, patients in group C received either 2 further cycles of T-DM1 + P (subgroup C1, responders) or were switched to 4 cycles of 5-fluorouracil + epirubicin + cyclophosphamide (FEC) (subgroup C2, non-responders) (Online Resource 1).

The primary endpoint of the Neo-peaks study was the rate of pCR (comprehensive pCR [CpCR] ypN0 [ypT0-TisypN0], including residual ductal carcinoma in situ), determined by central histopathological review. Responders were defined as patients with ≥ 30% shrinkage of the primary tumor at its longest diameter, as determined by magnetic resonance imaging, and with a Ki67 level of ≤ 10% or the absence of cancer cells on core needle biopsy.

### Surgery and postoperative treatment

Breast surgery was carried out within 10 weeks of completion of neoadjuvant therapy. Postoperative treatment was in accordance with the policy at each institution. The protocol recommended that trastuzumab be administered for ≥ 1 year, combining the preoperative treatment period (including the period when T-DM1 was administered) and the postoperative treatment period. For patients who achieved pCR, adjuvant chemotherapy was permitted if considered appropriate by the attending physician. In cases of residual invasive tumor, appropriate chemotherapy regimens such as anthracycline-based regimens were added according to the attending physician’s discretion. In patients with ER + disease, standard hormone therapy was administered for ≥ 5 years. Local radiotherapy (including regional lymph nodes) was administered as needed.

### Follow-up and endpoints

The following were evaluated at 6 months and at 1, 2, 3, 4, and 5 years after surgery (± 1 month): metastasis or recurrence, development of secondary cancer, survival, and adverse events.

In the present study, disease-free survival (DFS), distant DFS (DDFS), and overall survival (OS) were analyzed. DFS was defined as the period from the date of registration to death from any cause, recurrence of primary breast cancer, or a secondary cancer event. DDFS was defined as the period from the date of registration to diagnosis of distant metastasis of the primary cancer. OS was defined as the period from the date of registration to death from any cause.

For exploratory purposes, the results were analyzed with patients stratified by pCR achievement status after neoadjuvant therapy and by ER status. Additionally, patients in group C were divided into responders (C1) and non-responders (C2) based on the response determined after four cycles of the study treatment; the data were analyzed with the patients stratified by pCR achievement status and by use of anthracycline therapy.

### Safety

Adverse events were recorded in accordance with National Cancer Institute Common Terminology Criteria for Adverse Events (CTCAE version 4.03), version 4.0 (Japanese Clinical Oncology Group edition) [10].

### Ethical considerations

The study was conducted in compliance with the Declaration of Helsinki and relevant ethical guidelines for clinical and epidemiological studies. Approval from an ethics committee was obtained at each institution at the start of both the Neo-peaks study and the present follow-up study. Informed consent to participate in the Neo-peaks study was obtained from all patients at the time of enrollment. For the present follow-up study, data were collected from the medical records of the patients.

### Statistical analysis

In the analysis of DFS, DDFS, and OS, the Kaplan–Meier method was used to estimate survival curves, and the log-rank test was used for intergroup comparisons. In the analysis of treatment effects, the Cox proportional hazards model was used to calculate hazard ratios (HRs) and their 95% confidence intervals (CIs) for each group. Statistical analyses were carried out using SAS version 9.4 (SAS Institute Inc., Cary, NC, USA).

## Results

From August 2014 to February 2016, 204 patients (51, 52, and 101 in groups A–C, respectively) were randomized. For the present follow-up study, the data cut-off date was September 30, 2021, and the data were fixed on February 6, 2022.

Results for the primary endpoint of the Neo-peaks study (i.e., pCR rate after neoadjuvant therapy) are summarized in Online Resource 2. pCR rate was higher in group B (71.2%) than in groups A and C (in which pCR rates were similar to each other: 56.9 and 57.4%, respectively). Within group C, pCR rates were 62.5 and 38.1% in groups C1 and C2, respectively. By ER status, in groups A–C, respectively, pCR rates were 76.2, 73.9, and 66.7% in the ER-negative (ER–) cohort, and 43.3, 69.0, and 50.8% in the ER + cohort. Thus, in the latter cohort, pCR rate was significantly higher in group B than in groups A and C (*p* = 0.047 and *p* = 0.013, respectively).

### Patient disposition and characteristics

**Fig. 1:**
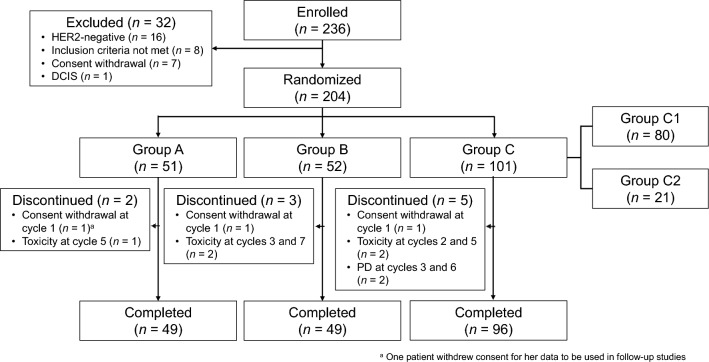
Patient disposition. DCIS, ductal carcinoma in situ; HER2, human epidermal growth factor receptor 2; PD, progressive disease

Figure 1 shows the patient flow; of the 236 patients enrolled, 204 were randomly allocated to the treatment groups. One patient in group A withdrew consent for the use of her data in the present follow-up study. In groups B and C, 3 and 5 patients, respectively, discontinued the study treatment in the neoadjuvant phase, however, maintained consent for use of data for follow up. Therefore, data for 203 patients (50, 52, and 101 patients in groups A–C, respectively) were analyzed. Table 1 summarizes the patients’ characteristics.

**Table 1:** Characteristics of patients included in the Neo-peaks study

Character	All (*n* = 203)	Group A (*n* = 50)	Group B (*n* = 52)	Group C (*n* = 101)	Group C1^a^ (*n* = 80)	Group C2^a^ (*n* = 21)
Age, years						
< 65	180 (88.7)	45 (90.0)	46 (88.5)	89 (88.1)	71 (88.8)	18 (85.7)
≥ 65	23 (11.3)	5 (10.0)	6 (11.5)	12 (11.9)	9 (11.3)	3 (14.3)
Median (range)	53 (25–70)	53 (28–70)	53 (29–69)	52 (25–70)	51.5 (25–70)	53 (40–67)
T: primary tumor						
T1c	44 (21.7)	11 (22.0)	13 (25.0)	20 (19.8)	14 (17.5)	6 (28.6)
T2	143 (70.4)	36 (72.0)	35 (67.3)	72 (71.3)	58 (72.5)	14 (66.7)
T3	16 (7.9)	3 (6.0)	4 (7.7)	9 (8.9)	8 (10.0)	1 (4.8)
N: regional lymph node						
N0	128 (63.1)	33 (66.0)	31 (59.6)	64 (63.4)	49 (61.3)	15 (71.4)
N1	75 (36.9)	17 (34.0)	21 (40.4)	37 (36.6)	31 (38.8)	6 (28.6)
Histological grade (B&R grade)						
1	4 (2.0)	0 (0.0)	2 (3.8)	2 (2.0)	1 (1.3)	1 (4.8)
2	64 (31.5)	13 (26.0)	19 (36.5)	32 (31.7)	23 (28.8)	9 (42.9)
3	62 (30.5)	17 (34.0)	14 (26.9)	31(30.7)	24 (30.0)	7 (33.3)
Unknown	73 (36.0)	20 (40.0)	17 (32.7)	36 (35.6)	32 (40.0)	4 (19.0)
Lymph node metastasis after surgery						
Did not undergo surgery	0 (0.0)	0 (0.0)	0 (0.0)	0 (0.0)	0 (0.0)	0 (0.0)
pN0	186 (91.6)	47 (94.0)	48 (92.3)	91 (90.1)	74 (92.5)	17 (81.0)
pN( +)	14 (6.9)	2 (4.0)	4 (7.7)	8 (7.9)	5 (6.3)	3 (14.3)
Unknown	2 (1.0)	1 (2.0)	0 (0.0)	1 (1.0)	0 (0.0)	1 (4.8)
Response to neoadjuvant therapy (CpCRypN0)						
Yes	124 (61.1)	29 (58.0)	37 (71.2)	58 (57.4)	50 (62.5)	8 (38.1)
No	78 (38.4)	21 (42.0)	15 (28.8)	42 (41.6)	29 (36.3)	13 (61.9)
Unknown	1 (0.5)	0 (0.0)	0 (0.0)	1 (1.0)	1 (1.3)	0 (0.0)
Surgical procedure						
Did not undergo surgery	1 (0.5)	0 (0.0)	0 (0.0)	1 (1.0)	1 (1.3)	0 (0.0)
Breast-conserving surgery	105 (51.7)	26 (52.0)	27 (51.9)	52 (51.5)	44 (55.0)	8 (38.1)
Total mastectomy	97 (47.8)	24 (48.0)	25 (48.1)	48 (47.5)	35 (43.8)	13 (61.9)
Axillary dissection procedure						
Did not undergo surgery	3 (1.5)	1 (2.0)	0 (0.0)	2 (2.0)	1 (1.3)	1 (4.8)
Axillary dissection	70 (34.5)	13 (26.0)	21 (40.4)	36 (35.6)	28 (35.0)	8 (38.1)
Axillary sampling dissection	20 (9.9)	6 (12.0)	4 (7.7)	10 (9.9)	7 (8.8)	3 (14.3)
SLN biopsy	105 (51.7)	29 (58.0)	27 (51.9)	49 (48.5)	42 (52.5)	7 (33.3)
SLN before therapy	5 (2.5)	1 (2.0)	0 (0.0)	4 (4.0)	2 (2.5)	2 (9.5)
Postoperative radiotherapy						
Breast-conserving surgery (*n* = 105)						
Yes (with regional lymph node irradiation) (*n* = 105)	18 (8.9)	4 (8.0)	9 (17.3)	5 (5.0)	4 (5.0)	1 (4.8)
Yes (without regional lymph node irradiation)	85 (41.9)	22 (44.0)	17 (32.7)	46 (45.5)	39 (48.8)	7 (33.3)
No	2 (1.0)	0 (0.0)	1 (1.9)	1 (1.0)	1 (1.3)	0 (0.0)
Total mastectomy (*n* = 97)						
Yes (with regional lymph node irradiation)	18 (8.9)	4 (8.0)	4 (7.7)	10 (9.9)	6 (7.5)	4 (19.0)
Yes (without regional lymph node irradiation)	3 (1.5)	0 (0.0)	1 (1.9)	2 (2.0)	1 (1.3)	1 (4.8)
No	76 (37.4)	20 (40.0)	20 (38.5)	36 (35.6)	28 (35.0)	8 (38.1)
Adjuvant chemotherapy						
Anthracycline						
Yes	48 (23.6)	5 (10.0)	4 (7.7)	39 (38.6)	18 (22.5)	21 (100.0)
No	155 (76.4)	45 (90.0)	48 (92.3)	62 (61.4)	62 (77.5)	0 (0.0)
Endocrine therapy						
Yes	117 (57.6)	27 (54.0)	29 (55.8)	61 (60.4)	46 (57.5)	15 (71.4)
No	85 (41.9)	23 (46.0)	23 (44.2)	39 (38.6)	33 (41.3)	6 (28.6)
Unknown	1 (0.5)	0 (0.0)	0 (0.0)	1 (1.0)	1 (1.3)	0 (0.0)
ER status before treatment (central assessment)						
Negative	86 (42.4)	21 (42.0)	23 (44.2)	42 (41.6)	36 (45.0)	6 (28.6)
Positive	117 (57.6)	29 (58.0)	29 (55.8)	59 (58.4)	44 (55.0)	15 (71.4)
HER2 status before treatment start						
IHC3 +	176 (86.7)	44 (88.0)	45 (86.5)	87 (86.1)	70 (87.5)	17 (81.0)
IHC2 + DISH +	27 (13.3)	6 (12.0)	7 (13.5)	14 (13.9)	10 (12.5)	4 (19.0)

Data are presented as *n* (%)

*B&R* Bloom and Richardson, *DISH* dual in situ hybridization, *ER* estrogen receptor, *HER2* human epidermal growth factor receptor 2, *IHC* immunohistochemistry, *SLN* sentinel lymph node

^a^Patients in group C were divided into subgroup C1, comprising patients who responded to the study treatment (i.e., 4-cycle T-DM1 + P), including those who withdrew during the study period; and subgroup C2, comprising patients who did not respond to the study treatment and were switched to an anthracycline-based regimen

### Long-term outcomes

**Fig. 2:**
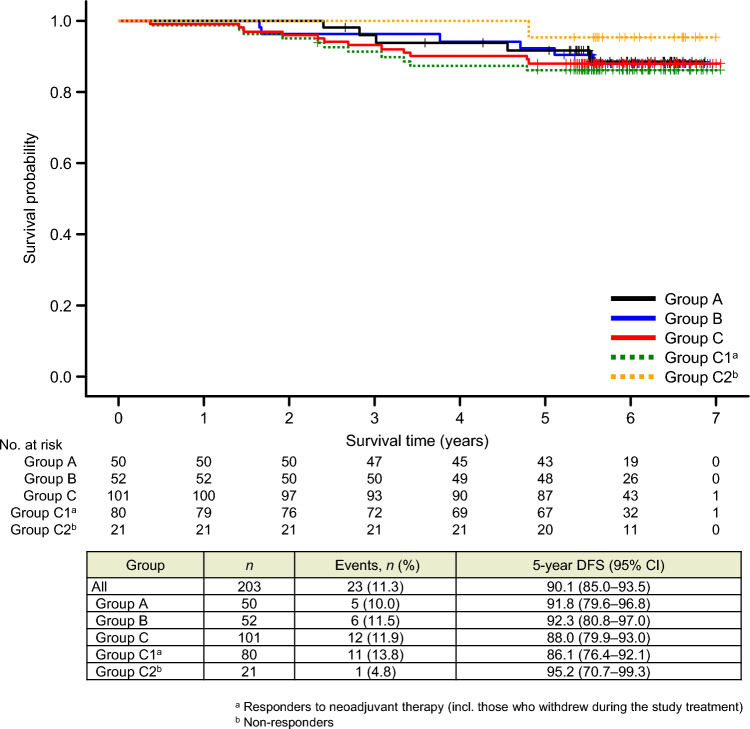
Kaplan–Meier curves for disease-free survival (DFS). Patients in group C were divided into subgroup C1, comprising patients who responded to the study treatment (i.e., 4-cycle T-DM1 + P), including those who withdrew during the study period; and subgroup C2, comprising patients who did not respond to the study treatment and were switched to an anthracycline-based regimen. *CI* confidence interval; *HR* hazard ratio

The 5-year DFS rates (95% CI) were 91.8% (79.6–96.8%), 92.3% (80.8–97.0%), and 88.0% (79.9–93.0%) in groups A–C, respectively. No significant differences in DFS were found among the treatment groups or subgroups (Fig. 2). DFS events occurred in 23 (11.3%) of the 203 patients; details are provided in Table 2. There were no cases of non-invasive relapse, so it was not included in the DFS analysis.

**Table 2:** Disease-free survival events (*n* = 23) in patients included in the Neo-peaks study, stratified by pathological complete response (pCR) status

Group	Locoregional		Distant		Secondary malignancy	
	*n*	Site of recurrence	*n*	Site of recurrence	*n*	Site of recurrence
*pCR achieved*						
A	1	Breast, ipsilateral	1	Brain	1	Stomach
B	0		1	Bone, cervical lymph nodes	0	
C1	1	Breast, ipsilateral	1	Liver	4	Contralateral breast (2) Pancreas Pleural mesothelioma
C2	0		0		0	
*pCR not achieved*						
A	0		1	Lung	1	Colon
B	0		2	Lung Bone	3	Contralateral breast Uterine body Lung
C1	2	Breast, ipsilateral Skin at the wound edge	3	Brain Lung with regional lymph node Mediastinal lymph node	0	
C2	0		0		1	Contralateral breast

pCR is defined as CpCRypN0, indicating absence of residual invasive tumor in the breast and evidence of lymph node metastasis on sentinel node biopsy and/or dissection carried out after systemic treatment

Data are presented as the number of patients and the type of disease event

**Fig. 3:**
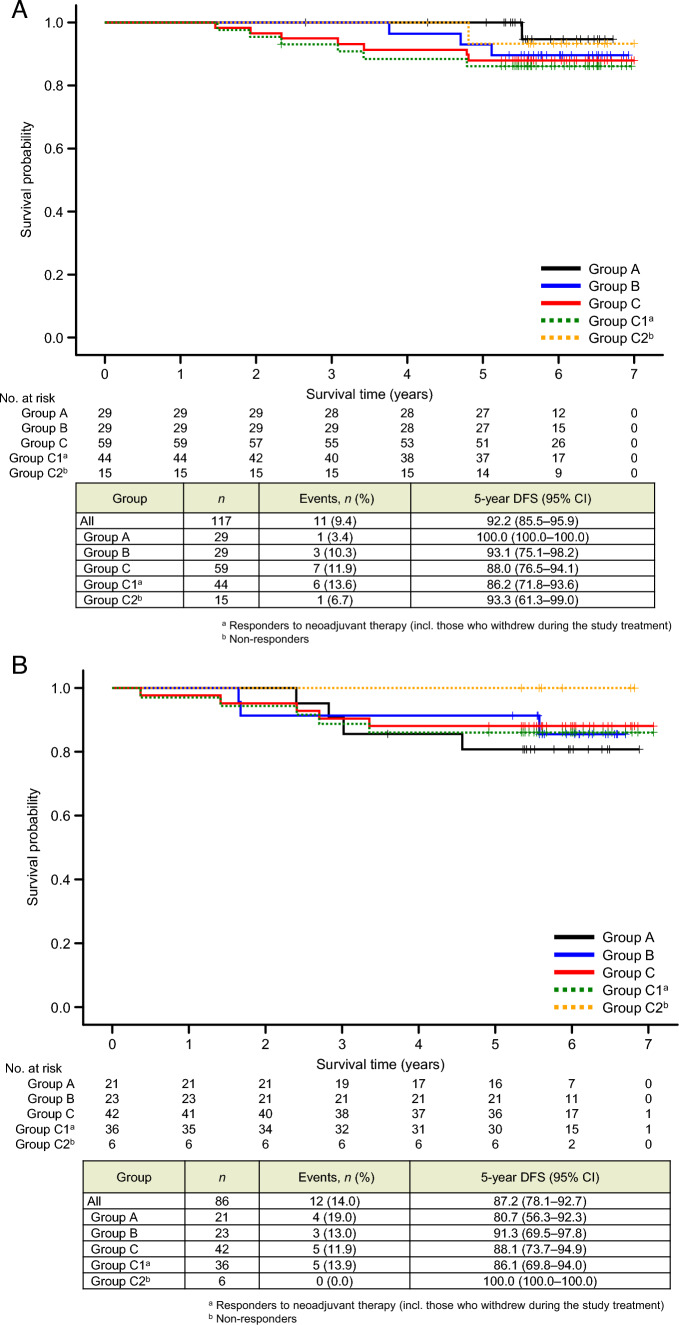
Kaplan–Meier curves for disease–free survival (DFS) in patients with estrogen receptor (ER)-positive disease (**A**), ER-negative disease (**B**). Patients in group C were divided into subgroup C1, comprising patients who responded to the study treatment (i.e., 4-cycle T-DM1 + P), including those who withdrew during the study period; and subgroup C2, comprising patients who did not respond to the study treatment and were switched to an anthracycline-based regimen. *CI* confidence interval; *HR* hazard ratio

Regarding DFS in patients with ER + or ER– disease (Figs. 3A and 3B, respectively), no significant intergroup differences were observed. However, DFS tended to be longer in patients with ER + disease.

**Fig. 4:**
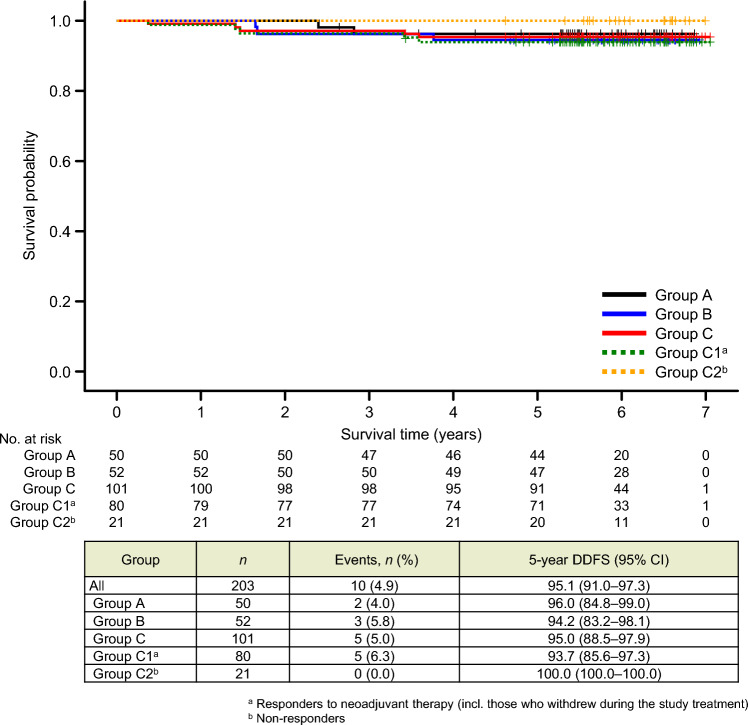
Kaplan–Meier curves for distant disease–free survival (DDFS). Patients in group C were divided into subgroup C1, comprising patients who responded to the study treatment (i.e., 4-cycle T-DM1 + P), including those who withdrew during the study period; and subgroup C2, comprising patients who did not respond to the study treatment and were switched to an anthracycline-based regimen. *CI* confidence interval; *HR* hazard ratio

**Fig. 5:**
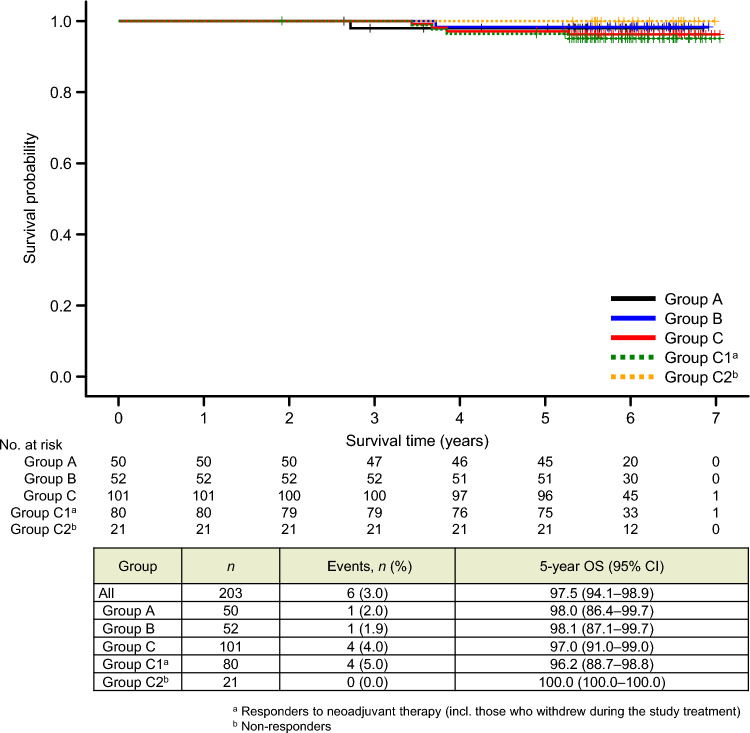
Kaplan–Meier curves for overall survival (OS). Patients in group C were divided into subgroup C1, comprising patients who responded to the study treatment (i.e., 4-cycle T-DM1 + P), including those who withdrew during the study period; and subgroup C2, comprising patients who did not respond to the study treatment and were switched to an anthracycline-based regimen. *CI* confidence interval; *HR* hazard ratio

There were also no significant differences in DDFS or OS (Figs. 4 and 5, respectively) for any comparison among the treatment groups and subgroups.

Overall, six deaths were recorded: 1 in group A, 1 in group B, and 4 in subgroup C1. There were no deaths in subgroup C2. Four deaths were due to breast cancer, and 2 deaths in subgroup C1 were due to other causes (1 due to an unknown cause and 1 due to secondary cancer).

#### pCR and long-term outcomes

**Fig. 6:**
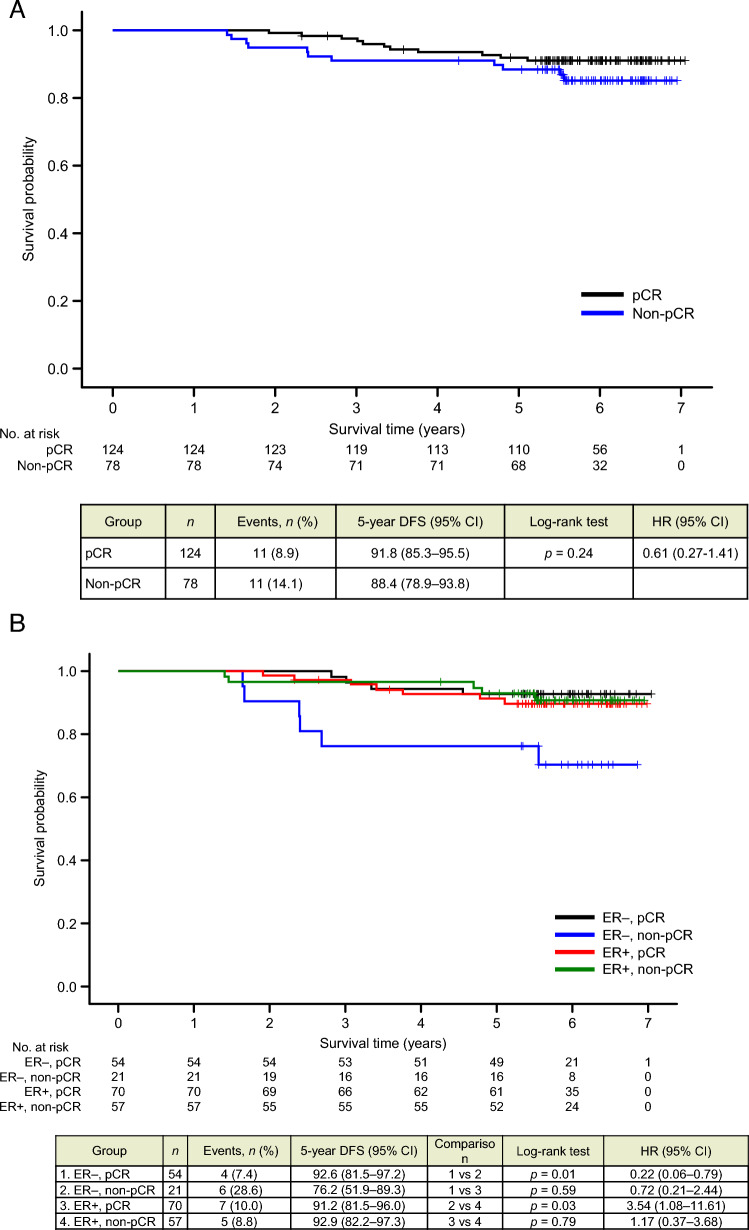
Kaplan–Meier curves for disease-free survival (DFS): data stratified by **A** pathological complete response (pCR) achievement status and **B** pCR and estrogen receptor (ER) status combined. *CI* confidence interval; *HR* hazard ratio

Figure 6A shows the results of analysis of DFS data stratified by pCR achievement status after neoadjuvant therapy. Patients who achieved pCR (*n* = 124) tended to have longer DFS than patients without pCR (*n* = 78), although the difference was not significant.

Figure 6B shows the results of analysis of data stratified by both pCR achievement and ER status. In the ER + subgroup, there was no significant difference in 5-year DFS between patients with and without pCR (*n* = 70 and *n* = 57, respectively; *p* = 0.79; HR, 1.17 [95% CI 0.37–3.68%]). By contrast, in the ER– subgroup, 5-year DFS was significantly longer in patients with pCR than in those without it (*n* = 54 and *n* = 21, respectively; *p* = 0.01; HR, 0.22 [95% CI 0.06–0.79%]).

#### Use of anthracycline therapy and long-term outcomes in group C

**Fig. 7:**
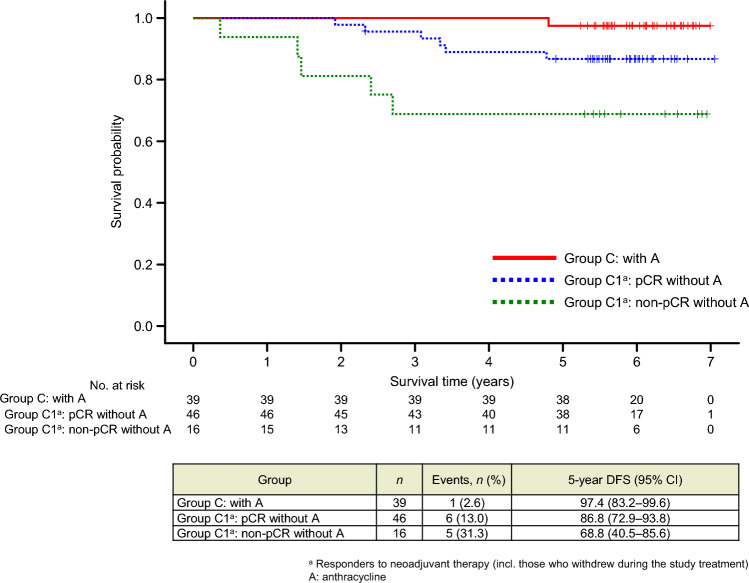
Kaplan–Meier curves for disease-free survival (DFS) in group C: curves for group C patients who received adjuvant anthracycline (**A**) (*n* = 39), and subgroup C1 patients who achieved pathological complete response (pCR) (*n* = 46) and those who did not (*n* = 16) after neoadjuvant treatment and did not receive adjuvant anthracycline. Patients in group C were divided into subgroup C1, comprising patients who responded to the study treatment (i.e., 4-cycle T-DM1 + P), including those who withdrew during the study period; and subgroup C2, comprising patients who did not respond to the study treatment and were switched to an anthracycline-based regimen. *CI* confidence interval

Patients in group C received 4 cycles of T-DM1 + P, and subsequently, either two further cycles of T-DM1 + P (subgroup C1, responders; *n* = 80) or four cycles of FEC (subgroup C2, non-responders; *n* = 21) as neoadjuvant therapy. In subgroup C1, four of the 50 patients who achieved pCR and 14 of the 30 patients who did not achieve pCR received adjuvant anthracycline. In subgroup C2, all 21 patients received neoadjuvant anthracycline; 8 patients achieved pCR and 13 did not. In groups C1 and C2 combined, a total of 39 patients received neoadjuvant or adjuvant anthracycline. Among these anthracycline recipients, DFS events occurred in only one patient who did not achieve pCR (subgroup C2), and the 5-year DFS rate was 97.4% (95% CI 83.2–99.6%) (Fig. 7, red line). In subgroup C1, 62 patients (46 with pCR and 16 with non-pCR) did not receive adjuvant anthracycline. Among these non-recipients of anthracycline, the 5-year DFS rate was 86.8% (95% CI 72.9–93.8%) for pCR patients and 68.8% (95% CI 40.5–85.6%) for non-pCR patients (Fig. 7; blue and green lines, respectively). In the former group (i.e., pCR patients who did not receive adjuvant anthracycline; *n* = 46), there was a single event of locoregional recurrence (ipsilateral breast tumor) and of distant recurrence (liver), and 6 events were secondary malignant diseases (Table 2). As shown in Table 3, which shows the DFS, DDFS, and OS results for group C and subgroup C1, among the non-pCR patients in subgroup C1, DFS was significantly better in patients who received adjuvant anthracycline than in those who did not (5-year DFS rate, 100% versus 68.8%, respectively; *p* = 0.02).

**Table 3:** Disease-free survival (DFS), distant disease–free survival (DDFS), and overall survival (OS) in group C patients included in the Neo-peaks study

Group	*n*	DFS		DDFS		OS	
		Events, *n* (%)	5-year rate (95% CI)	Events, *n* (%)	5-year rate (95% CI)	Events, *n* (%)	5-year rate (95% CI)
C: with A^a^	39	1 (2.6)	97.4 (83.2–99.6)	0	100 (100–100)	0	100 (100–100)
C1: pCR without A	46	6 (13.0)	86.8 (72.9–93.8)	1 (2.2)	97.8 (85.6–99.7)	2 (4.3)	97.8 (85.6–99.7)
C1: non-pCR with A	14	0	100 (100–100)	0	100 (100–100)	0	100 (100–100)
C1: non-pCR without A	16	5 (31.3)	68.8 (40.5–85.6)	4 (25.0)	75.0 (46.3–89.8)	2 (12.5)	86.7 (56.4–96.5)

*A* anthracycline; *pCR* pathological complete response

^a^Patients in group C were divided into subgroup C1, comprising patients who responded to the study treatment (i.e., 4-cycle T-DM1 + P), including those who withdrew during the study period; and subgroup C2, comprising patients who did not respond to the study treatment and were switched to an anthracycline-based regimen. This row shows data from the total of 39 patients who received anthracycline in the perioperative period (21 patients in group C2 who received anthracycline as neoadjuvant therapy; 4 pCR patients and 14 non-pCR patients in subgroup C1 who received anthracycline after surgery)

### Safety

During the follow-up period, there were no unexpected drug-related adverse events, and no treatment discontinuations or deaths due to adverse events.

## Discussion

The aims of the Neo-peaks study were to investigate the usefulness of the new treatment approach of adding T-DM1 + P to the conventional TCbHP regimen, and to test the potential non-inferiority of the entire chemotherapy-free regimen (i.e., T-DM1 + P) compared with the TCbHP regimen. Additionally, in the group of patients who received the chemotherapy-free regimen, the possibility of treatment adjustment (i.e., addition of anthracycline therapy) after neoadjuvant T-DM1 + P regimen was examined. This was an exploratory analysis of prognosis 5 years after surgery.

In the present follow-up study, no significant differences were found between patients who received only TCbHP (6 cycles) (group A) and those who received TCbHP (4 cycles) followed by T-DM1 + P (4 cycles) (group B) in terms of DFS, DDFS, or OS, regardless of ER status, although pCR rate after neoadjuvant therapy tended to be higher in group B than in group A, and the difference was significant in the ER + subgroup [8].

Because pCR rate does not directly reflect patient benefits and is considered a surrogate endpoint rather than a true endpoint, it is important to evaluate long-term outcomes, which should be regarded as true endpoints. In the present study, the improvement in pCR rate due to addition of T-DM1 + P to TCbHP did not translate to differences in the long-term. Therefore, the current standard TCbHP regimen (6 cycles) remains a recommended option as neoadjuvant therapy for HER2 + primary breast cancer.

In the present study, we also investigated a chemotherapy-free T-DM1 + P regimen followed by response-guided treatment adjustment. Not only pCR rate but also long-term outcomes (DFS, DDFS, and OS) were similar in patients who received this treatment (group C) and in those who received TCbHP (group A). We conclude that it may be feasible to use the chemotherapy-free T-DM1 + P regimen followed by response-guided treatment adjustment. This de-escalating approach would benefit patients considerably by eliminating the adverse effects associated with chemotherapy. However, to determine more appropriate strategies, for example based on identification of patients requiring rescue treatment and appropriate rescue treatment regimens, further studies are needed.

We found that patients who responded to neoadjuvant T-DM1 + P and subsequently completed the full six cycles (i.e., subgroup C1) were more likely to achieve pCR than non-responders (i.e., subgroup C2). However, long-term outcomes, in terms of DFS, DDFS, and OS, tended to be longer in the non-responders. Because non-responders to neoadjuvant therapy are generally considered to have poor prognosis, the results presumably reflect the beneficial treatment effects conferred by the use of anthracycline therapy in this group.

In group C (*n* = 101), patients were categorized as responders or non-responders after completion of the first four cycles. This approach contributed to the 62.5% pCR rate achieved in responders (*n* = 80), which was equivalent to that achieved with the standard TCbHP regimen (used to treat group A) [8]. In non-responders, for whom anthracycline was added to their treatment, a pCR rate of 38.1% (8/21 patients) was achieved [8]. Additionally, their 5-year prognosis was similar to that of patients who received the standard TCbHP regimen (i.e., group A), possibly due to the use of anthracycline after response-guided treatment adjustment.

The results of the phase 3, randomized, open-label KAITLIN study (NCT01966471) showed that T-DM1 + P (18 cycles) was not superior to the standard taxane + trastuzumab + pertuzumab (THP) regimen (three or four cycles), each administered after three or four cycles of anthracycline-based chemotherapy, when used as adjuvant therapy in the treatment of high-risk HER2-positive early stage breast cancer [11]. The 3-year invasive disease–free survival (IDFS) rates were similar in the THP and T-DM1 + P groups: 94.2 and 93.1%, respectively (HR, 0.98 [95% CI 0.72–1.32]). In the Neo-peaks study, the use of T-DM1 + P was response-guided. Using this approach, both pCR and long-term prognosis were similar to those of the standard TCbHP regimen (although interpretation of these results is limited due to the small sample size). The number of T-DMI + P cycles in the Neo-peaks study was lower than that in the KAITLIN study, which suggests the possibility of treatment de-escalation.

KRISTINE was a randomized phase 3 study comparing 6 cycles of T-DM1 + P against TCbHP as neoadjuvant therapy in patients with HER2 + breast cancer (*n* = 444) [7]. Patients who received the T-DM1 + P regimen had a significantly lower pCR rate and shorter event-free survival [12]. By contrast, our results suggest that by using a response-tailored approach, pCR rate and long-term prognosis similar to those achieved with TCbHP may be possible. Therefore, in the preoperative period, appropriate treatment adjustment may be necessary in order to develop less toxic, de-escalated treatment.

In the present study, for patients for whom T-DM1 was ineffective (i.e., group C2), we considered rescue therapy with anthracyclines to be possibly useful, because the mechanism of action of these drugs (mainly inhibition of DNA topoisomerase II) differs from that of drugs used in the prior treatments (emtansine, the cytotoxic payload of T-DM1, inhibits tubulin polymerization, and taxane anticancer drugs, e.g., docetaxel and paclitaxel, are also tubulin inhibitors). Anthracycline-free rescue treatment regimens such as TCHP (docetaxel, carboplatin, trastuzumab, and pertuzumab) may also be useful for non-responders such as those in group C2.

In subgroup C1, the 18 patients who received adjuvant anthracycline had no DFS events, whereas DFS events occurred in 11 of the 62 patients who did not receive adjuvant anthracycline. Notably, among the 30 patients who did not achieve pCR after neoadjuvant T-DM1 + P, DFS was significantly worse in the 16 patients who did not receive adjuvant anthracycline than in the 14 patients who did. This finding indicates that adjuvant anthracycline should be used to treat non-pCR patients after neoadjuvant T-DM1 + P. In the 46 patients who achieved pCR and did not receive adjuvant anthracycline, 6 DFS events occurred; of these, four were secondary malignant diseases. Based on the small number and the rarity of events, it is difficult to reach conclusions for these patients, and further studies are necessary to identify patients who require adjuvant chemotherapy.

Regarding potential safety concerns, in the phase 3 APHINITY trial (*n* = 4769) [13], dual blockade with trastuzumab + pertuzumab (HP) was found to carry no greater cardiac risk than trastuzumab alone (incidence of cardiac events: 3.5% versus 3.2%, respectively). Anthracycline use was associated with increased cardiac risk; therefore, non-anthracycline chemotherapy may be appropriate for patients with cardiovascular risk factors. Further investigation is needed regarding non-anthracycline drugs for use in adjuvant chemotherapy.

To date, various neoadjuvant drug therapies for treatment of HER2 + early breast cancer have been investigated in terms of pCR rate and long-term outcomes. Standard neoadjuvant treatment regimens include TCbHP and doxorubicin plus cyclophosphamide (AC) followed by THP. Several studies have been conducted to investigate optimal regimens as well as to explore de-escalating approaches (in which chemotherapy is omitted to reduce toxicity).

TRYPHAENA was a randomized phase 2 study comparing TCbHP (6 cycles) and FEC (3 cycles) followed by THP (3 cycles) as neoadjuvant therapy in patients with HER2 + early breast cancer (*n* = 225). The results showed similar pCR rates and DFS in all treatment arms [14, 15].

The WSG-ADAPT-HER2 + /HR– trial was a randomized phase 2 study comparing a chemotherapy-free regimen with trastuzumab + pertuzumab (HP) against THP in patients with HER2 + , HR-negative early breast cancer (*n* = 134) in a neoadjuvant setting [16, 17]. At 12 weeks, pCR rate was significantly higher in the THP arm than in the HP arm; however, no significant differences were observed in terms of IDFS, DDFS, or OS at 5 years [17]. Furthermore, the 5-year results suggested that further chemotherapy could safely be omitted in patients who have achieved pCR [17].

In the phase 2 WSG-ADAPT-TP study, T-DM1, T-DM1 and hormone therapy, and trastuzumab and hormone therapy were compared in a 12-week neoadjuvant setting in patients with HER2 + , HR–positive early breast cancer [18]. Although pCR rate was significantly lower in patients who received trastuzumab plus hormone therapy, no significant differences were observed among the three treatment groups in terms of IDFS or OS [19]. The study also showed that among 117 patients with pCR, DFS was similar between in patients who received adjuvant chemotherapy and in those who did not.

Among all patients in the present study, pCR rates tended to be lower but DFS tended to be longer in patients with ER + disease than in those with ER– disease; this is consistent with the results previously reported [20]. In the present study, in the ER– subgroup, DFS was significantly longer in patients who achieved pCR than in those who did not, whereas in the ER + subgroup, DFS was similar regardless of whether pCR had been achieved. These findings may be due to the positive effects of adjuvant hormone therapy that patients with ER + disease received after surgery. The usefulness of addition of hormone therapy to T-DM1 has also been evaluated in the WSG-ADAPT-TP study [18, 19]. The results of that study showed that addition of hormone therapy to T-DM1 is unlikely to substantially influence pCR, IDFS, or OS.

The usefulness of another novel ADC, trastuzumab deruxtecan (T-DXd), is currently being investigated in several ongoing trials. In DESTINY-Breast05 (ClinicalTrials.gov identifier, NCT04622319), it is being evaluated as adjuvant therapy versus T-DM1 in high-risk patients who did not achieve pCR after neoadjuvant therapy [21]. In DESTINY-Breast11, T-DXd is being evaluated as monotherapy or followed by THP versus the standard AC followed by THP [22]. Chemotherapy-free neoadjuvant T-DXd is also being investigated, and its use with response-guided treatment adjustment is yet to be explored. Additionally, in DESTINY-Breast09, a combination of T-DXd plus pertuzumab is being evaluated as a first-line treatment for metastatic breast cancer [23]. The feasibility of this regimen as perioperative treatment remains to be explored.

### Limitations

The present study has several limitations. First, the number of patients may be insufficient to determine the long-term prognosis. In particular, in patients with ER + disease, the risk of long-term recurrence after 5 years has not been evaluated. Lastly, the study was conducted before adjuvant therapy with T-DM1 became standard treatment.

## Conclusions

The three neoadjuvant therapy regimens investigated in the present study, namely TCbHP, TCbHP followed by T-DM1 + P, and T-DM1 + P (response guided), resulted in similar long-term outcomes. Our results support the current standard neoadjuvant TCbHP, although T-DM1 + P with response-guided treatment adjustment may serve as a potential treatment option when there are reasons to minimize toxicity. Chemotherapy is recommended for non-pCR patients after ADC-based neoadjuvant treatment. However, based on the observation in the present study of distant recurrence affecting 1 (2.2%) of 46 patients, it may be possible to cautiously consider omitting anthracycline from treatment for some patients who have achieved pCR, provided the potential benefit (less toxicity) is judged to outweigh the potential risk (loss of chemotherapeutic treatment effects).

### Supplementary Information

Below is the link to the electronic supplementary material.

Supplementary file1 (DOCX 20 KB)

Supplementary file2 (PPTX 215 KB)

## Data Availability

Data available on request due to privacy/ethical restrictions.
