# Supplementary material for: Long-term outcomes of neoadjuvant trastuzumab emtansine + pertuzumab (T-DM1 + P) and docetaxel + carboplatin + trastuzumab + pertuzumab (TCbHP) for HER2-positive primary breast cancer: results of the randomized phase 2 JBCRG20 study (Neo-peaks)
Source: Breast Cancer Res Treat. 2024 May 20;207(1):33–48. doi: 10.1007/s10549-024-07333-7 (PMC11230995; doi:10.1007/s10549-024-07333-7)
Supplement: Supplementary file 1 — Supplementary file1 (DOCX 20 KB) [file 10549_2024_7333_MOESM1_ESM.docx]

**Online Resource 2**

**Table** Pathological complete response (pCR)^a^ rate in patients included in the Neo-peaks study after neoadjuvant therapy

| Patient category (*n*) | pCR rate, % | *p* |
| --- | --- | --- |
| Group A (51) | 56.9 |  |
| Group B (52) | 71.2 |  |
| Group C (101) | 57.4 |  |
| Group C1 (80)^b^ | 62.5 |  |
| Group C2 (21)^b^ | 38.1 |  |
| *Stratified by ER status* |  |  |
| Group A |  |  |
| ER-negative (21) | 76.2 |  |
| ER-positive (30) | 43.3 |  |
| Group B |  |  |
| ER-negative (23) | 73.9 |  |
| ER-positive (29) | 69.0 | 0.047 (vs group A, ER-positive), 0.013 (vs group C, ER-positive) |
| Group C |  |  |
| ER-negative (42) | 66.7 |  |
| ER-positive (59) | 50.8 |  |
| Group C1^b^ |  |  |
| ER-negative (36) | 72.2 |  |
| ER-positive (44) | 54.5 |  |
| Group C2^b^ |  |  |
| ER-negative (6) | 33.3 |  |
| ER-positive (15) | 40.0 |  |
| *Stratified by HER2 expression status* |  |  |
| Group A |  |  |
| IHC3+ (45) | 57.8 |  |
| IHC2+ DISH+ (6) | 50.0 |  |
| Group B |  |  |
| IHC3+ (45) | 73.3 |  |
| IHC2+ DISH+ (7) | 57.1 |  |
| Group C |  |  |
| IHC3+ (87) | 64.4 | < 0.01 (vs group C, IHC2+ DISH+) |
| IHC2+ DISH+ (14) | 14.3 |  |
| Group C1^b^ |  |  |
| IHC3+ (70) | 68.6 | < 0.01 (vs group C1, IHC2+ DISH+) |
| IHC2+ DISH+ (10) | 20.0 |  |
| Group C2^b^ |  |  |
| IHC3+ (17) | 47.1 |  |
| IHC2+ DISH+ (4) | 0.0 |  |

*ER* estrogen receptor, *DISH* dual color in situ hybridization, *HER2* human epidermal growth factor receptor 2, *IHC* immunohistochemistry

^a^ Defined as CpCRypN0, indicating absence of residual invasive tumor in the breast and evidence of lymph node metastasis on sentinel node biopsy and/or dissection carried out after systemic treatment

^b^ Patients in group C were divided into subgroup C1, comprising patients who responded to the study treatment (i.e. 4-cycle T-DM1+P), including those who withdrew during the study period; and subgroup C2, comprising patients who did not respond to the study treatment and were switched to an anthracycline-based regimen
