# Supplementary figures and images for: Long-term outcomes of neoadjuvant trastuzumab emtansine + pertuzumab (T-DM1 + P) and docetaxel + carboplatin + trastuzumab + pertuzumab (TCbHP) for HER2-positive primary breast cancer: results of the randomized phase 2 JBCRG20 study (Neo-peaks)
Source: Breast Cancer Res Treat. 2024 May 20;207(1):33–48. doi: 10.1007/s10549-024-07333-7 (PMC11230995; doi:10.1007/s10549-024-07333-7)

## Slide 1
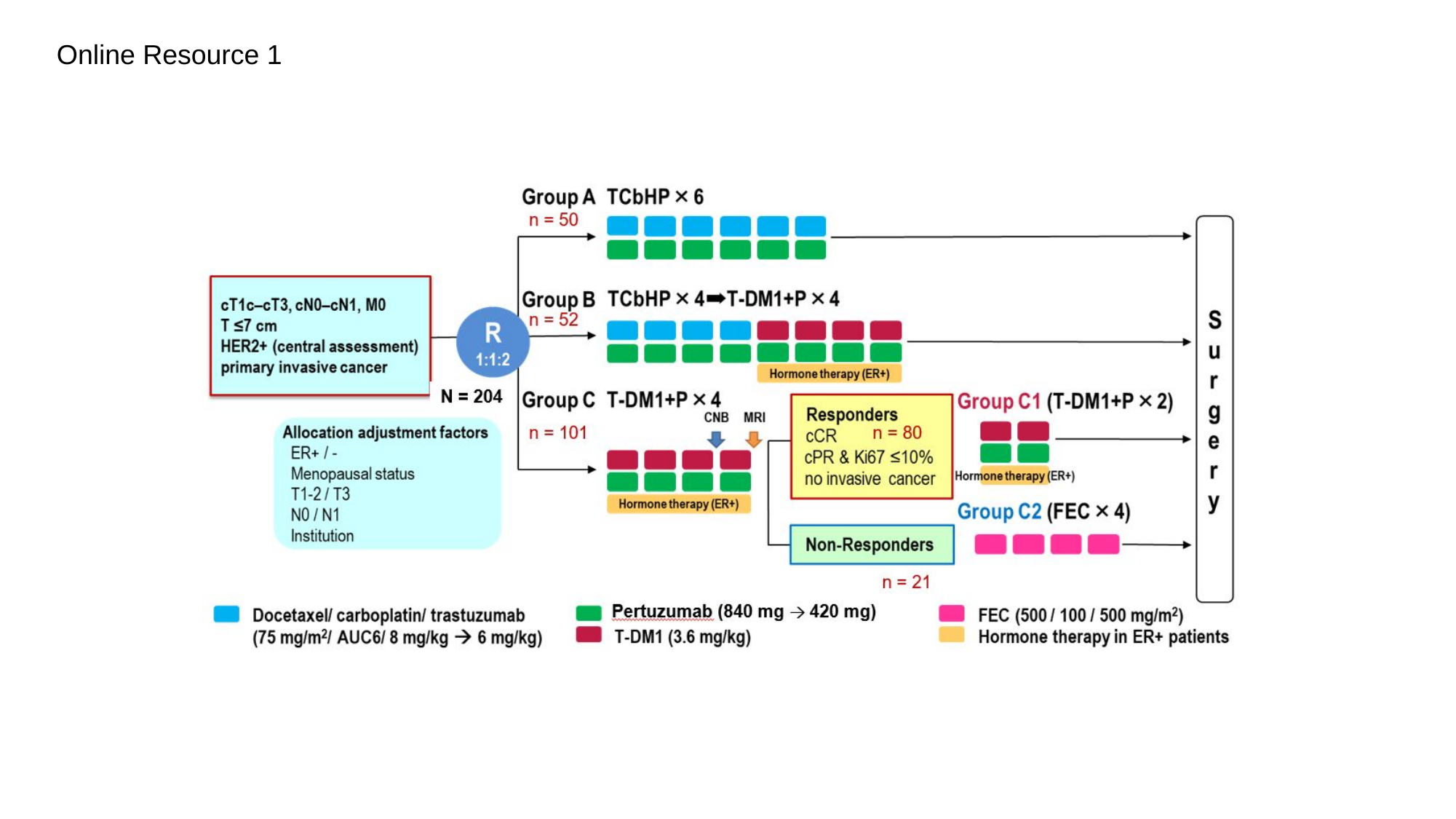

Online Resource 1

Supplement: Supplementary file 2 — Supplementary file2 (PPTX 215 KB) [file 10549_2024_7333_MOESM2_ESM.pptx]
